# Uncommon Presentation of Tuberculosis as an Incidentally Discovered Solitary Pleural Tuberculoma

**DOI:** 10.4269/ajtmh.21-0740

**Published:** 2021-10-25

**Authors:** Takeshi Kinjo, Mitsuyoshi Shimoji, Jiro Fujita

**Affiliations:** ^1^ Department of Infectious, Respiratory and Digestive Medicine, Graduate School of Medicine, University of the Ryukyus, Okinawa, Japan;; ^2^ Department of Thoracic and Cardiovascular Surgery, Nanbu Tokushukai Hospital, Okinawa, Japan

**Figure 1.**
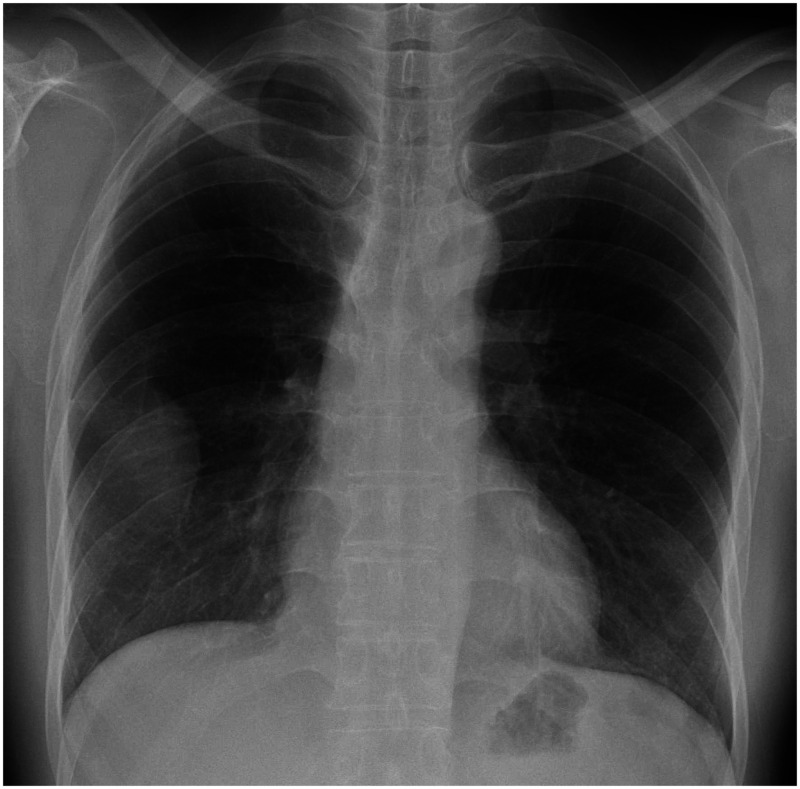
Incomplete border sign on chest X-ray. A mass was observed in the right lower lung field. Note that the outline of the mass disappeared on the right side (incomplete border sign), suggesting an extra-pulmonary lesion.

**Figure 2.**
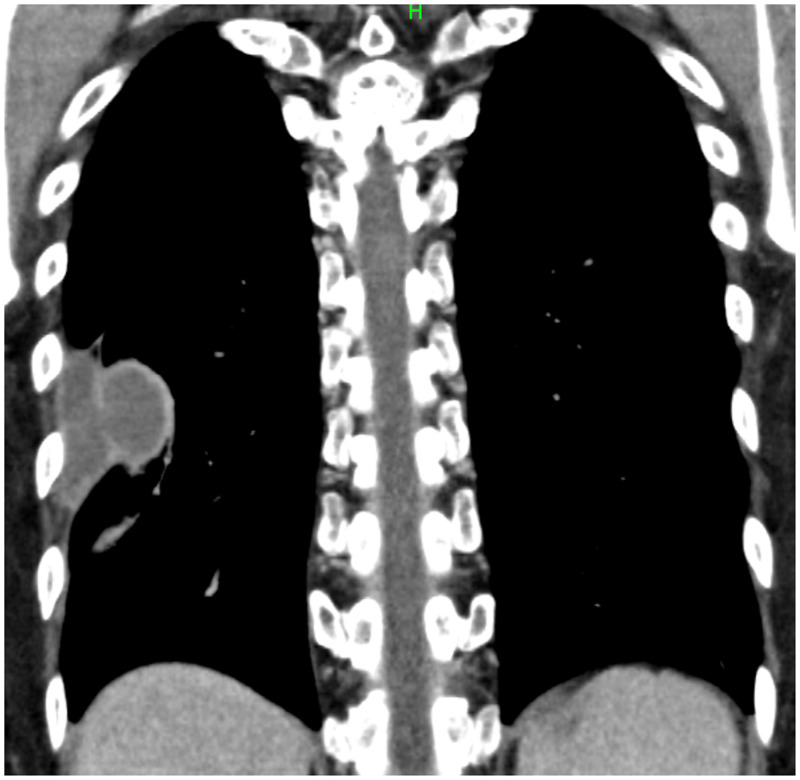
Coronal section of enhanced chest computed tomography. Encapsulated and peripherally enhanced low-attenuation mass adjacent to the posterolateral chest wall was observed. Note that the lower outline of the mass connects smoothly to the chest wall (extra-pleural sign), suggesting extra-pulmonary lesions. Pleural effusion was not detected.

**Figure 3.**
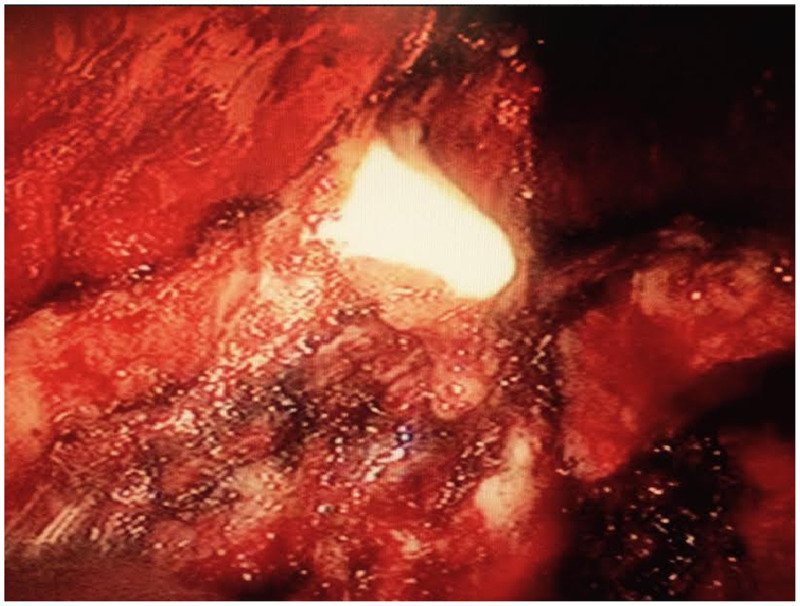
Intrathoracic view during surgery. There was a discharge of white viscous pus from the mass. This figure appears in color at www.ajtmh.org.

A 54-year-old Japanese man with unremarkable social and medical history presented with an abnormal shadow on his chest X-ray during an annual company health checkup. He had the bacille Calmette-Guérin vaccination as a child and had no smoking/foreign travel history. He was asymptomatic, with normal vital signs. He had a white blood cell count of 6,610 cells/µL and a C-reactive protein level of 0.11 mg/dL. Chest X-ray revealed a mass with an incomplete border sign in the right lower lung field (Figure 1). Contrast-enhanced chest computed tomography revealed an encapsulated and peripherally enhancing low-attenuation mass adjacent to the posterolateral chest wall that exhibited an extra-pleural sign (Figure 2). No pleural effusion was observed. Because a chest wall tumor was suspected, video-assisted thoracoscopic surgery for tumor resection was performed. During the surgery, the mass discharged white viscous pus (Figure 3); a specimen was collected, which later tested positive for *Mycobacterium tuberculosis* complex using a real-time polymerase chain reaction assay targeting 16S recombinant RNA (Cobas TaqMan MTB; Roche Diagnostics, Basel, Switzerland). Pathological examination revealed granuloma formation with central caseous necrosis, indicating tuberculosis (TB). Although bacterial culture of the pus for acid-fast bacilli was negative, an additional blood test was positive for interferon-γ release assay. Because there was no other comorbidity, such as pulmonary TB, the patient was diagnosed with solitary pleural tuberculoma and received antitubercular agents for 6 months (2 months of isoniazid, rifampicin, ethambutol, and pyrazinamide, followed by 4 months of isoniazid, rifampicin, and ethambutol). The abnormal shadow on the patient’s chest X-ray disappeared completely during the course of treatment and did not recur for at least for 2 years after completion of the antituberculous therapy. A detailed interview revealed that his sister suffered from pulmonary TB when he was 1 year old.

Pleural tuberculoma usually occurs during chemotherapy against pleural or pulmonary TB, possibly as a result of a paradoxical or hypersensitivity reaction to killed *M. tuberculosis*.^1^^,^^2^ Pleural tuberculoma without associated effusion or antituberculous therapy is rare. Furthermore, solitary pleural tuberculoma, as shown in our patient case, is extremely rare, with only one reported case in literature.^3^ The pathophysiology of pleural tuberculoma in our patient might be explained by the reactivation of *M. tuberculosis* in an adjacent pleural lymph node originally infected during the phase of primary TB infection.^4^ This rare disease should be considered as a cause of a pleural mass, even in patients who are not receiving antituberculous therapy. In addition, this case emphasized the value of detecting classical signs on chest X-ray.^5^
